# Navigating the Pedagogical Landscape: Exploring the Implications of AI and Chatbots in Nursing Education

**DOI:** 10.2196/52105

**Published:** 2024-06-13

**Authors:** Muthuvenkatachalam Srinivasan, Ambili Venugopal, Latha Venkatesan, Rajesh Kumar

**Affiliations:** 1 College of Nursing All India Institute of Medical Sciences Mangalagiri India; 2 College of Nursing All India Institute of Medical Sciences New Delhi India; 3 College of Nursing All India Institute of Medical Sciences Rishikesh India

**Keywords:** AI, artificial intelligence, ChatGPT, chatbots, nursing education, education, chatbot, nursing, ethical, ethics, ethical consideration, accessible, learning, efficiency, student, student engagement, student learning

## Abstract

This viewpoint paper explores the pedagogical implications of artificial intelligence (AI) and AI-based chatbots such as ChatGPT in nursing education, examining their potential uses, benefits, challenges, and ethical considerations. AI and chatbots offer transformative opportunities for nursing education, such as personalized learning, simulation and practice, accessible learning, and improved efficiency. They have the potential to increase student engagement and motivation, enhance learning outcomes, and augment teacher support. However, the integration of these technologies also raises ethical considerations, such as privacy, confidentiality, and bias. The viewpoint paper provides a comprehensive overview of the current state of AI and chatbots in nursing education, offering insights into best practices and guidelines for their integration. By examining the impact of AI and ChatGPT on student learning, engagement, and teacher effectiveness and efficiency, this review aims to contribute to the ongoing discussion on the use of AI and chatbots in nursing education and provide recommendations for future research and development in the field.

## Introduction

Artificial intelligence (AI) and AI-based chatbots such as ChatGPT have become popular in many industries, including health care. AI refers to the use of computer algorithms and machine learning techniques to enable machines to perform tasks that traditionally require human intelligence, such as perception, reasoning, and decision-making. AI has numerous applications in health care, including in nursing education. AI uses computers and specially designed software to perform tasks and reasoning in different areas of health care, including screening, diagnosis, education, telecommunications, data security, finance, research, and the legal system [1,2]. However, in the present era, the scope of AI is limited to carrying out specific tasks and solutions to predefined problems [3].

A chatbot is a computer program designed to simulate conversation with human users through text or voice-based interactions. Chatbots use natural language processing and AI technologies to understand and respond to user queries and requests in a conversational manner [4]. ChatGPT is one such state-of-the-art language processing models that generates human-like responses to text prompts. It is trained on vast amounts of text data and uses machine learning algorithms to predict the likelihood of a particular sequence of words. OpenAI, a Microsoft Corp–backed start-up, recently unveiled GPT-4, a highly advanced version of ChatGPT. GPT-4’s upgraded capabilities enable it to engage in dialogic conversations and provide more comprehensive answers, incorporating improved data, facts, and analytical insights. It predicts the next word in a given set of text based on patterns it learned from a massive amount of data during its training process. The AI processes user requests and responds based on available information. Likewise, AI is identical to machine learning and human learning and uses specially designed software and machine-based algorithms to complement human learning in education, learning, analysis, and other multifaceted medical and health care fields [5].

The scope of this viewpoint paper is to explore the pedagogical implications of AI and AI-powered chatbots in nursing education; it will examine the potential uses and benefits of AI and chatbots in nursing education and the challenges and ethical considerations that must be addressed when integrating these technologies into the classroom. By examining the impact of AI and chatbots on student learning, engagement, and teacher effectiveness and efficiency, this viewpoint paper aims to provide educators and researchers in nursing education with a deeper understanding of the potential benefits and challenges of integrating AI and chatbots into their teaching practice.

However, there are also challenges and ethical considerations that must be addressed. As such, it is crucial to examine the role of AI and chatbots in nursing education and explore its implications to prepare nursing students for the digital age. The objective of this viewpoint paper is to provide insights into navigating the pedagogical landscape of nursing education with AI and chatbots.

## Understanding AI and Chatbots in Nursing Education

Chatbots have potential applications in health care education, research, and practice [6]. There is currently a surge in the development of desktop and mobile applications powered by AI and ChatGPT technology. Several chatbots have been created with specialized capabilities to perform specific tasks. These specialized chatbots are designed to excel in their specific area of expertise and are trained with data and algorithms that enable them to provide accurate and relevant responses to user queries related to their area of specialization.

In nursing education, AI and chatbots can potentially transform the learning experience for students and teachers alike. For example, ChatGPT can be used to summarize large amounts of text data such as research articles, clinical notes, and patient records, which could help nurses quickly identify key findings and insights from a large body of literature [7]. Chatbots help nursing students interact with AI-generated natural language prompts to better understand medical concepts. It has the potential to become a go-to assistant for nursing students who aspire to become more proficient in their field. Students can use chatbots to develop study schedules, create multifaceted questions and scenarios, and quiz themselves on topics they want to be prompted about. Recent developments have been made in this area, such as ChatGPT being integrated as a journal author for a publication titled, “Open Artificial Intelligence Platforms In Nursing Education: Tools For Academic Progress Or Abuse” [8]. AI can be used to analyze data and identify patterns to personalize learning material and provide student feedback. It can also be used to simulate patient scenarios and provide a safe and controlled environment for students to practice clinical skills. Chatbots using AI technology can also provide immediate responses to students’ questions and offer 24/7 support, enabling students to learn at their own pace and on their own schedule.

Additionally, AI can be used to improve the efficiency and effectiveness of administrative tasks such as scheduling, record-keeping, and grading. This can free up teachers’ time to focus on more meaningful student interactions, such as providing personalized feedback and support. The potential uses of AI and chatbots in nursing education are vast and varied, offering exciting opportunities for innovation and transformation in the field.

## Benefits of AI and Chatbots in Nursing Education

Incorporating AI and chatbots in nursing education leads to a multitude of benefits, including personalized learning experiences achieved by analyzing data and identifying patterns. This tailoring of learning materials offers customized feedback based on each student’s needs, which, in turn, enhances engagement and motivation. AI-powered simulations provide a safe environment for practicing clinical skills, thus minimizing risks to real patients, while AI-driven chatbots, such as ChatGPT, offer continuous support and immediate responses to questions, thus promoting self-paced learning [9]. Furthermore, AI streamlines administrative tasks, such as grading and record-keeping, allowing educators to focus on meaningful student interactions. Real-time risk assessment and triage of patients are facilitated by machine learning–based systems such as Enlitic, which prioritize and direct cases to appropriate clinicians [10]. The influence of AI and chatbots on nursing education manifests in improved student learning and engagement. Interactional learning experiences, such as simulated patient scenarios and gamification, foster enhanced engagement and outcomes. Additionally, AI and chatbots improve access to resources by providing round-the-clock support, fostering a culture of continuous learning.

## Pedagogical Implications of Integrating AI Into Nursing Education

The integration of AI and chatbots into nursing education brings forth several pedagogical implications, including increased engagement and motivation for students through personalized feedback, adaptation to individual learning styles, and prompt responses to queries. This enhanced learning experience improves students’ knowledge retention and problem-solving skills by offering real-time feedback, tailored assessments, and interactional simulations. Additionally, AI and chatbots augment teacher support by streamlining grading, feedback, and administrative tasks, allowing educators to concentrate on more meaningful interactions with their students. Finally, the development of clinical competencies and confidence is facilitated by simulating realistic patient scenarios coupled with immediate feedback, better preparing students for encounters with actual patients in the clinical setting.

## Teaching Effectiveness and Efficiency

AI and chatbots can significantly enhance teaching effectiveness and efficiency in nursing education in various ways. These technologies enable more efficient grading and feedback, reducing the workload for educators and offering students immediate feedback for faster learning and better retention. An AI-powered writing assistant can check student papers for grammar, spelling errors, plagiarism, and readability, providing instant feedback and allowing teachers to focus on more in-depth evaluations. Moreover, AI and chatbots can facilitate personalized teaching by adapting to individual learning styles and identifying knowledge gaps, resulting in improved learning outcomes. Adaptive learning systems can assess students’ strengths and weaknesses, tailoring teaching materials to their specific needs.

Furthermore, AI and chatbots can create realistic simulations for students to practice clinical skills and build confidence in their abilities. Virtual reality simulations can provide lifelike patient scenarios for practicing skills such as medication administration or vital sign monitoring. Additionally, these technologies can augment teacher support by identifying struggling students and offering targeted interventions to enhance their learning outcomes. Learning analytics tools can help educators track student progress and pinpoint areas requiring additional support.

## Integrating AI Into Nursing Education for Clinical Excellence

The integration of AI into nursing education marks a transformative stride toward clinical excellence. As AI technologies become increasingly prevalent in health care, nurses are presented with new opportunities and challenges. This paradigm shift prompts a reevaluation of nursing education practices, emphasizing the need for a curriculum that incorporates AI competencies [11]. In a hospital setting, training on AI-assisted patient monitoring systems for nursing nurses may equip them to interpret AI-generated insights, enabling proactive interventions. For example, nurses using AI alerts to predict deteriorating patient conditions can respond swiftly, showcasing the interdependence of human expertise and AI assistance in ensuring patient safety. Nurse educators play a pivotal role in this transformation, guiding students to navigate AI applications within clinical settings. This holistic approach seeks to bridge the gap between traditional nursing practices and the advancements brought forth by AI, ultimately shaping a future where clinical excellence is synonymous with technological proficiency.

## Limitations

Despite the significant benefits of incorporating AI and chatbots in nursing education, there are essential limitations to consider. One critical aspect is the lack of human interaction, as AI and chatbots cannot entirely replace the development of interpersonal skills and empathy in nursing students. Ethical considerations, such as privacy, confidentiality, and bias, must be thoroughly addressed to ensure the safe, effective, and ethical use of these technologies without harming patients or perpetuating inequalities.

The adoption of AI and chatbots in nursing education necessitates a significant financial investment. Institutions must allocate resources for acquiring advanced technologies, specialized software, and ongoing technical support. Moreover, the costs associated with staff training on these technologies should not be underestimated. This financial burden can be prohibitive for some institutions, potentially creating disparities in access to AI-enhanced education [12]. Another concern is the potential for error, as AI and chatbots are not infallible and may lead to incorrect diagnoses or treatment plans, requiring cautious use to avoid misdiagnoses in medical tests. By carefully considering and addressing these limitations, the integration of AI and chatbots into nursing education can be optimized for safety, effectiveness, and ethicality.

## Ethical and Social Considerations

Although AI and chatbots offer numerous benefits to nursing education, they also bring about ethical and social concerns that must be considered [13]. Issues such as bias and discrimination, privacy and security, accountability and transparency, displacement of human labor, and dehumanization and depersonalization must be addressed [14]. For example, biased or incomplete data used to train AI systems could lead to skewed information on certain health conditions or patient populations. Biased or incomplete data used to train AI systems in nursing education may manifest in skewed information on specific health conditions or patient populations. For instance, let us consider an AI-driven module designed to teach nursing students about prevalent health issues in India. If the training data predominantly include information from urban health care settings, the AI system may unintentionally neglect health concerns prevalent in rural areas. In this scenario, the AI system, having learned from biased data, might emphasize urban-centric health challenges while overlooking issues specific to rural communities, such as unique infectious diseases or limited access to certain health care resources. As a result, nursing students exposed primarily to this skewed information may not be adequately prepared to address the diverse health needs of the entire population, leading to an unintentional bias in their education. To mitigate this, it is essential to ensure that the training data encompass a comprehensive representation of health care scenarios, including both urban and rural contexts. Thus, the AI system can offer a more balanced and inclusive educational experience, fostering a nuanced understanding of diverse health conditions prevalent across different regions of India.

In addition, sensitive data storage and processing raise privacy and security concerns, while the opaque nature of AI systems presents challenges for accountability and transparency. To address these considerations, it is vital to develop and use AI and chatbot systems ethically and responsibly. This includes ensuring data diversity and representativeness, implementing robust privacy and security measures, promoting transparency and accountability, and creating policies and regulations to tackle potential social and economic implications. Involving stakeholders, such as educators, students, and patient advocates, in the development and implementation of AI and chatbot systems is essential to align them with ethical and social values, fostering the best possible outcomes for nursing education [15].

## Challenges and Opportunities

Integrating AI and chatbots into nursing education can present significant challenges and barriers for nursing educators. Technical expertise and resources, such as programming skills and access to specialized hardware and software, may be required to integrate these technologies into teaching and learning practices successfully. Additionally, some educators and students may resist change, and nursing programs may need additional training and support to encourage adoption. The cost of implementing AI and chatbots may be prohibitive, and programs must carefully consider the long-term sustainability of such investments. Data quality and availability may also be challenging, as AI and chatbots require large amounts of data to be effective.

Concerns related to data theft and cybersecurity are the major challenges, as these technologies are vulnerable to breaches and unauthorized access to confidential patient information. Adherence to laws and guidelines concerning information technology use and implementation of stringent security measures is crucial to address this issue [16]. Additionally, incorporating AI and chatbots requires technical support, infrastructure, and expertise, which may be challenging for institutions with limited resources or technical capabilities. Since AI models and platforms are often developed by professionals outside the nursing and medical fields, end users such as nurses and health care professionals may struggle to understand the technical aspects, potentially leading to errors or inaccurate findings [17].

Although laden with challenges, integrating AI and chatbots into nursing education, presents numerous opportunities and potential solutions. One key aspect is providing technical support and training to both educators and students, ensuring their comfort with these new technologies. Adopting a collaborative approach with other nursing programs and institutions can alleviate cost and sustainability issues, as resources, knowledge, and expertise can be shared among them. Moreover, forging partnerships with technology companies and industry experts can grant access to specialized hardware, software, and proficiency in AI and chatbots’ implementation.

Another crucial factor is establishing clear data governance policies and protocols to tackle data quality and availability challenges while securing sensitive information. Developing ethical frameworks and guidelines can help address ethical and legal concerns, including data privacy, security, bias, discrimination, and accountability. By incorporating AI and chatbots into innovative pedagogical approaches, nursing education can create more personalized learning experiences for students, leading to improved health care delivery. By capitalizing on these opportunities and implementing potential solutions, nursing programs can successfully integrate AI and chatbots, reaping the numerous benefits and opportunities offered by these advanced technologies.

## Best Practices for Integrating AI and Chatbots in Nursing Education

Integrating AI and chatbots in nursing education requires a strategic approach that considers the unique needs and goals of each institution. While some nursing programs have been slower in integrating AI and chatbots, others have successfully implemented these technologies to improve student learning outcomes and enhance teaching effectiveness. One such example is the use of virtual patient simulations, which use AI to create realistic patient scenarios for nursing students to practice their skills in a safe and controlled environment [18,19]. Another successful integration of AI in nursing education is the use of adaptive learning platforms, which use chatbots to provide personalized learning experiences for students based on their individual needs and learning styles. These platforms can help identify areas of weakness and provide targeted feedback to improve student learning outcomes. Additionally, some nursing programs have used AI-powered chatbots to provide 24/7 support to students, answering their questions and providing additional resources and support when needed. These examples demonstrate the potential benefits of integrating AI and chatbots in nursing education and outline best practices for other institutions looking to implement these technologies.

To ensure the effective and ethical integration of AI and chatbots in nursing education, a thoughtful approach and best practices are essential. It is crucial to define clear goals that align with the institution’s mission and educational objectives, fostering targeted and effective use of AI and chatbots for improved student learning outcomes. Involving key stakeholders, such as nursing faculty, educational technologists, and student representatives, in the planning process guarantees their needs and concerns are considered.

Adequate training and support for faculty and students are crucial for the successful integration of AI and chatbots into the curriculum, ensuring their effective use in enhancing learning outcomes. Continual evaluation and refinement of AI and chatbots’ implementation, based on data regarding their impact on student learning outcomes, enables institutions to make informed decisions about future adjustments.

As AI and chatbots transform nursing education, further research and development are needed to maximize their potential benefits. With increased integration, ethical considerations such as privacy, bias, and transparency must be tackled to ensure equitable use. For instance, AI algorithms could perpetuate health care biases if not properly designed and validated. Future research should explore ways to mitigate these biases, promoting social justice and equity in AI and chatbots’ usage.

Another area for future research is the long-term impact of AI and chatbots on nursing education. While early studies suggest that these technologies can enhance student learning outcomes and teaching effectiveness [20], it is important to evaluate their long-term impact on the nursing profession. Future research can explore how integrating AI and chatbots in nursing education affects the nursing workforce and their ability to provide quality patient care.

## Conclusion

The viewpoint paper discusses the various ways AI and chatbots can be used in nursing education, such as providing personalized learning experiences, facilitating clinical reasoning, and enhancing communication skills. Additionally, the review highlights the potential implications of these technologies on the nursing profession, including improving patient outcomes, advancing research, and promoting evidence-based practice. The impact of AI and chatbots on nursing education and the nursing profession as a whole can be significant. These technologies can provide nursing educators new tools to engage students and enhance their learning experiences. Moreover, they can help nurses improve their clinical practice and provide better patient care.

AI and chatbots have the potential to revolutionize nursing education and the nursing profession. Educators and researchers in nursing education should explore the potential of these technologies and incorporate them into their teaching practices. However, addressing the potential ethical implications of using AI in nursing education and practice is also important. Therefore, researchers and educators should collaborate to develop guidelines and best practices to ensure the responsible use of AI in nursing education and practice.

## Abbreviations

AI: artificial intelligence
